# Structure Life Extension towards the Structural Integrity of Sukhoi Su-30MKM

**DOI:** 10.3390/ma14195562

**Published:** 2021-09-25

**Authors:** Arvinthan Venugopal, Roslina Mohammad, Md Fuad Shah Koslan, Ashaari Shafie, Alizarin Ali, Owi Eugene

**Affiliations:** 1 Centre of Aerospace Engineering Services Establishment, Subang Airbase, Shah Alam 40000, Selangor, Malaysia; arvinthanvenugopal@yahoo.com (A.V.); md.fuad@airforce.mil.my (M.F.S.K.); 2 Razak Faculty of Technology and Informatics, Universiti Technology Malaysia, Jalan Sultan Yahya Petra, Kuala Lumpur 54100, Malaysia; 3 Aerospace Technology Systems Corporation Sdn Bhd, ATSC Corporate Centre, PT 192, Jalan Lapangan Terbang, Subang 47200, Selangor, Malaysia; ashaari@atsc.com.my; 4 CAIDMARK Sdn Bhd, Damansara Utama, Petaling Jaya 47400, Selangor, Malaysia; alizarin@caidmark.com.my; 5 RMAF Combat Training School, TUDM Bukit Ibam, Muadzam Shah 26700, Pahang, Malaysia; ovii90@gmail.com

**Keywords:** critical fatigue location, structure life extension, Su-30MKM, non-destructive testing, aircraft structural integrity program

## Abstract

The airframe structures of most fighter aircraft in the Royal Malaysian Airforce have been in service for 10 to 20 years. The effect of fatigue loading, operating conditions, and environmental degradation has led to the structural integrity of the airframe being assessed for its airworthiness. Various NDT methods were used to determine the current condition of the aircraft structure after operation of beyond 10 years, and their outcomes are summarized. In addition, although there are six critical locations, the wing root was chosen since it has the highest possibility of fatigue failure. It was further analyzed using simulation analysis for fatigue life. This contributes to the development of the maintenance task card and ultimately assists in extending the service life of the fighter aircraft. Using the concept of either safe life or damage tolerance as its fatigue design philosophy, the RMAF has adopted the Aircraft Structural Integrity Program (ASIP) to monitor the structural integrity of its fighter aircraft. With the current budget constraints and structural life extension requirements, the RMAF has embarked on the non-destructive testing method and engineering analysis. The research outcome will enhance the ASIP for other aircraft platforms in the RMAF fleet for its structure life assessment or service life extension program.

## 1. Introduction

A reliable and maintainable aircraft system is a mandatory requirement of any air force, with the Royal Malaysian Air Force (RMAF) being no exception. Airframe structures are the major system that require attention in terms of structural integrity. By definition, structural integrity is the ability of a structure to perform as the design intended in terms of structural safety, resilience, and dependability. A structure’s integrity is affected by the material’s mechanical properties under different thermal and environmental conditions. The need is to identify a permissible material fabrication and joining method and an economical in-service repair method soon.

NDT methods are beneficial in terms of the downtime maintenance period and cost effective. They can be introduced incrementally in the new servicing concept, such as plan schedule servicing (PSS) to replace progressive preventive and restoration work (PPRW) at 1000 h for MiG 29 (RMAF, 2007a) and progressive maintenance to replace the maintenance interval for Su-30MKM (RMAF, 2007b), which the RMAF has done. In addition, maintenance will be more precise by incorporating structural inspection methods using the non-destructive testing (NDT) to anticipate any structural and component failure. 

Structural and component failure can be categorized into fracture and no fracture, which can be due to various factors, such as thermal, mechanical, and chemical. Force can result from static or cyclic loads, which can cause mechanical failure.

Aircraft structures and components are inevitably subjected to static and cyclic loading and are indiscriminately prone to defect or crack initiation, ultimately leading to material fracture. Even though material fatigue has been widely studied over the years, with numerous test and experiment data collected, according to the National Aerospace Laboratories, Bangalore, India, [1] both metallic and non-metallic fatigue failures in aircraft structures and components still account for around 60% of total failures, as shown in Table 1.

Aircraft operators around the world experience fatigue damage, an example being the total loss of MB326H of the Royal Australian Air Force (RAAF) MB326 fighter lead-in jet trainer aircraft. An investigation revealed fatigue failure in the wing section [2]. In a similar event in 1969, two aircraft of the Royal Airforce (RAF) and one United States Air Force (USAF) F-111 fighter bomber faced catastrophic failure after just 100 h of flying.

**Table 1:** Frequency of failure modes in aircraft components [3].

Failure Mode	Percentage of Failures	
	Engineering Component	Aircraft Component
Corrosion	29	16
Fatigue	25	55
Brittle fracture	16	-
Overload (ductile)	11	14
High-temperature corrosion	07	02
Corrosion fatigue	06	07
Creep	03	-
Wear/abrasion/erosion	03	06

The USAF started implementing the Aircraft Structural Integrity Program (ASIP) after the incident of the B-47 crash in the year 1958 due to fatigue failure [4]. The USAF introduced the damage tolerance requirement as per MIL-A-8344 in the year 1976. The ASIP states a guideline for the design of new aircraft entering the service of the USAF to ensure that structural integrity is the main factor in the service life and implementation of the usage monitoring system.

One of the leading causes of fatigue failure in the RMAF is its surrounding tropical environment, besides the operational mission, and this leads to structural corrosion and corrosion-induced cracking [5]. Fatigue management in RMAF aircraft was only via documentation of flight hours and landing cycle throughout the fleet, as stipulated by the original equipment manufacturer (OEM). Said aircraft would be decommissioned and withdrawn from service after certain flight hours are completed. The cycle-counting method was a barrier to the advancement of science [6]. Systems such as peak count (minimum and maximum limits) have paved the way for the concept of fatigue meter, also known as the g-meter. This system collects the stress and strain data of the aircraft during service, and the OEM has introduced this approach for RMAF Hawk and Su-30MKM. 

The primary objective of usage monitoring in structural integrity management is to ensure that the life of type (LoT) of an aircraft approaches the planned withdrawal date of the aircraft, with the operating condition within the design envelope and limitations and without major structural damage beyond the safe-life design limit. However, as the aircraft approaches its operational life limit, fatigue failure is likely to occur. This principle is conservative, but analyses and predictions of the in-service fatigue life and cracks can increase the airworthiness of the aircraft. However, the inspection, detection, and repair of the damaged component remain dependent factors. 

With increasing operational life of an aircraft, fatigue-related failure starts to occur. The structure no longer assumes a perfect structural part, like the safe-life component, but rather assumes that the new part already has a defect that will eventually evolve, leading to catastrophic failure. This theory seems too conservative, but analysis and prediction of in-service fracture and cracking instances can increase the integrity of the aircraft structure to a higher level of airworthiness. However, it also depends on various factors, such as inspecting, repairing, or replacing the component and complete failure [7]. 

Implementing safe life in aircraft maintenance made a few countries execute the Service Life Extension Program (SLEP) for an aircraft’s extended service life span. In the context of the RMAF, the approach taken in the SLEP for Su-30MKM was to use the non-destructive testing (NDT) method and simulation analysis as a basis for the determination of the service life extension. The NDT defines the methods used to identify the damage in the aircraft’s fatigue-critical locations, while the simulation is performed for virtual fatigue testing to determine the fatigue-to-failure rate.

### 1.1. Summary of the Structure Life Extension Method

The Aircraft Structural Integrity Program (ASIP) approach has been integrated into the SLEP for Su-30MKM. Early detection of damage is crucial for maintaining safe structures, and non-destructive inspection is the key to ensuring that the structure operates safely for extended periods of service. The following Table 2 is the previous work done by various researchers on structure life extension programs: 

### 1.2. Problem Statement

- As it enters the twenty-first century, the RMAF faces three challenges in its mission to sustain a competent, state-of-the-art air force. The issues are increasing aircraft unit costs, reduced operating budget, and the continuous need for a force structure adequate to meet the nation’s pledges. Aircraft being operated commercially or by the military will eventually reach a stage, after a specific number of operation years, referred to as a structural life extension. In conjunction with their operating requirements, the Sukhoi aircraft will have to go through a service life structure assessment based on which of the following criteria is met first: 10 years’ service life calendar date
- 1500 airframe flying hours
- 5.25 million loading factors (calculated from Sukhoi’s ARM TSV System)

In the RAAF and USN F/A-18A/B fleet, a fatigue test is carried out to define the fatigue life and the danger point, such as the point of failure on a test piece exposed to a series of stress amplitude, which finally determines the aging factor for the specified aircraft. Idealizing the test settings would likely vary one or more factors affecting fatigue life [8]. However, although these conditions are satisfied, there will always be several unknown and uncontrollable causes that produce a large scatter in fatigue life. The Sukhoi aircraft is relatively new, having no retired parts on which to perform the actual fatigue test. Therefore, the non-destructive testing method is used for determining the fatigue-critical location and the factors contributing toward fatigue damage and validated through the simulation technique. Malaysia’s Sukhoi fleet entered service in 2009. The Su-30MKM fleet’s exact airframe hour for each aircraft is an RMAF-guarded secret, and this paper can only reveal that the average number of hours for the fleet is 864 flight hours, which is only 57% of the total 1500 flying hours required for the aircraft to be overhauled. This means the 10-year calendar date came first, in 2019 [9]. The service life structure assessment is compulsory to determine the Sukhoi aircraft’s airworthiness after a certain period. The Sukhoi’s 10-year servicing program was later renamed Sukhoi’s Progressive and Restoration Works (PRW) and was introduced to undertake the service life extension requirements. 

Due to budget constraints and the exorbitant cost quoted by the original equipment manufacturer (OEM) and because currently no maintenance manual is available for the 10-year structure life overhaul, the RMAF decided to perform the task according to its own capability and expertise. As the aircraft’s calendar time and flight time increase over the years, the effects of corrosion and cracking from fatigue and accidental damage are concerning factors in terms of its airworthiness. In addition, life extension requires continued airworthiness valuations of structural life extension activities. The Su-30MKM is a strategic asset, and its continuous existence is vital to protect the Malaysian airspace. After 10 years of operation, the aircraft is due for an overhaul. The RMAF aspired to manage the fleet, while using local capabilities for self-sustenance. Thus, it was decided to develop the maintenance and overhaul task card locally by implementing the non-destructive inspection and engineering analysis to ensure uninterrupted Su-30MKM operation. 

### 1.3. Fatigue Design Concept

The stress-life (S–N) method has been adopted extensively. This method relies on the principle that safe life is simply the accumulated damages subject to constant amplitude (CA) and variable amplitude (VA). Safe life is then scaled down using the scatter, depending upon engineering assessment and experiences.

**Figure 1:**
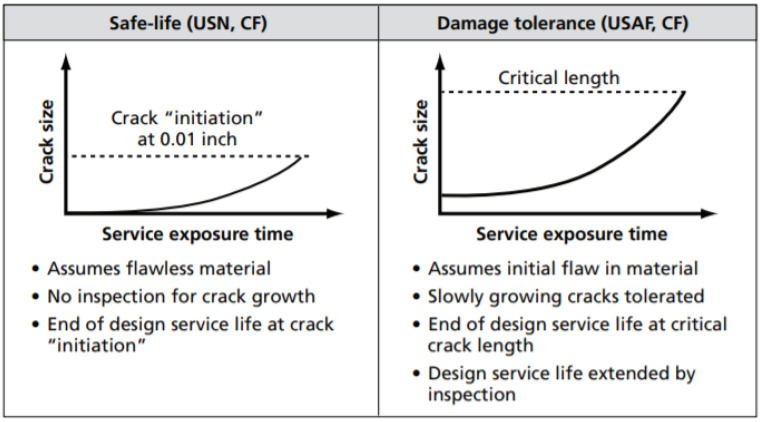
Safe life and damage tolerance concept [10].

Safe life and damage tolerance are two well-defined design principles in the aircraft structure against fatigue damage, and the two approaches are shown in Figure 1. Their damage growth modeling differentiates the two design principles. The aircraft design is based on the distinctive assumption of the initial material conditions and failure criteria. In contrast, the safe-life design is defined by assuming that no fatigue cracks exist on the aircraft structure throughout the safe operation in a specific life cycle. The damage tolerance approach is based on the assumption that fatigue cracks will potentially occur at fracture-critical parts. Conventionally, the safe-life approach still takes into consideration the existence of tiny cracks in the structure. The difference from the damage tolerance approach, which considers cracks that propagate slowly, is acceptable until the cracks can be detected and repaired. Operators worldwide do not follow one standard method of fatigue management since there are no particular specifications for this data collection. In the case of the Su-30MKM aircraft, it is built per the fatigue philosophy of safe life. This fatigue management process’s primary purpose is to incorporate a load-monitoring program on each aircraft to routinely measure the load’s cycle in its primary structure.

While military transport aircraft have rigorous mission profiles, fighters or attack-type aircraft are known to experience substantial variability in their missions. Therefore, they cannot be tracked based on mission hours alone, and based on the literature, an individual aircraft tracking (IAT) program is necessary for agile combat-type aircraft. The most significant benefit of this IAT program is that load monitoring can be performed even without any knowledge or information about the exact stress location. With a sufficient number of primary load-carrying structures routinely monitored, stresses at all critical locations can be determined.

The Royal Malaysian Airforce decided to adopt the safe-life approach in order to access its fighter aircraft. 

### 1.4. Structural Fatigue and Durability Criteria

Structural durability is considered as a quantitative measure of the resistance to initial fatigue cracking under specified conditions. This concept and the inspection-free requirements have led to the structural design philosophy of a safe-life aircraft for the Su-30MKM in which its service life is equal to 6000 FH. Although the RMAF is adopting the ASIP, which is in line with the MIL-STD-1530D, for the Su-30MKM, the ASIP is based on the safe-life concept. For fatigue analysis for its metallic structure and durability analysis, Miner’s rule was agreed on as the common method of damage accumulation. The generalized fatigue S–N curves used in the fatigue analysis are standard design curves agreed upon for the Su-30MKM structure analysis.

### 1.5. Structural Critical Location

The primary structure elements (PSEs), also known as the fatigue-/fracture-critical locations (FCLs), are components whose failure could compromise the aircraft’s airworthiness. The PSE can be made up of a single component or an assembly of 40–70 components. The PSE varies between aircraft. The FCL can be determined through stress analysis, fatigue testing, flight test, and design drawing. The structural critical location can be divided into two main categories: structure significant item (any load-bearing assembly whose failure could affect the structural integrity of the aircraft) and non-significant structure (defined both externally and internally within zonal boundaries). Therefore, the selection of the FCL of the aircraft structure should be carried out and strength, stiffness, and fatigue life estimation should be carried out [11].

### 1.6. Fighter Aircraft Mission Profile

**Figure 2:**
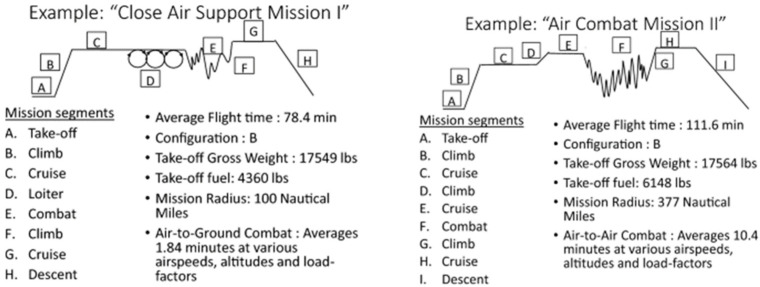
Representation of one mission profile [10].

A mission profile consists of combinations of events representing the loadings that a component or structure may experience during its life. Any mission profile, as shown in Figure 2, will specify the number of maximum maneuver turns or the number of turns at a particular altitude, Mach number, and power setting. Ten years of flight history (2008–2017) obtained from the ARM-TSV has been used to represent future usage analysis. The g-history is an example of compiled 2008 flight history obtained from the ARM-TSV workstation. These data were obtained from the flight data recorder (FDR) and compiled manually for each year. The original data were per flight mission and typically lasted up to 2–3 h, and the “rainflow cycle” counting procedure was then applied automatically on the g-loading profile spectrum.

The loading profile generally contains two blocks, and one block is equivalent to one year of flying. Each block (even number) assumes that the aircraft wing tip is fitted with the missile, and the other configuration block (odd number) assumes the wing is clean, without the missile. Each mission sortie is smoothened out in both profiles by omitting the smallest end loads to 15% of the total damage. In post-modification profiles, the peak and valley loads are lowered by 8%, with the number of cycles shortened. Fatigue life calculation requires:

- The spectrum loading (g-loading).
- Material properties, with fatigue characteristics, either S–N or E–N formulation.
- The component to be analyzed. The component should have actual physical geometry and should be analyzed to obtain its displacement, stress, and strain result.

Operating out of this mission profile would incur high cycle fatigue stress on the aircraft structure. Over the years, this could be catastrophic. The safe-life approach or the crack initiation method used the E–N (strain life) formulation. Furthermore, the low cycle fatigue (LCF) approach was adopted because the loading profile is the g-acceleration data.

The time stamp for each mission is in minutes:seconds (mm:ss), and during compilation, the time format was compiled in milliseconds only. The missions were compiled accordingly for each year (from the year 2008 until 2017). The missions were added one after the other according to the date each mission was flown.

### 1.7. Individual Aircraft Tracking

Fighter aircraft often operate in multiple roles, which translate into different operational loadings throughout the service life. Recognizing each aircraft’s overloads during service through individual aircraft tracking (IAT) is crucial. Knowledge of the aircraft structure loading is necessary to have access to the aircraft structure’s consumed fatigue life [12]. Even with the implementation of safe life, multiple operational roles cause the total rate of fatigue damage to be higher or lower than the predicted rate, and some aircraft are decommissioned at different flight hours.

The state-of-the-art onboard monitoring system enables the complex combination of loads to be recorded, which is the main driving force behind IAT [13]. Conventionally, the aircraft’s safe life is determined when the fleet average load factor (*N_z_*) approaches the design spectrum. However, newer-generation aircraft now operate in different operational roles, and beyond the design envelope, the average load spectrum of the fleet is assumed to be inaccurate. Furthermore, due to non-linear aerodynamic and adaptive controls, the wing root’s bending moment should be monitored instead of *N_z_* [14]. 

IAT enables real-time monitoring of stress and load of the main load-bearing structure and other critical locations. Another advantage of IAT is that the data collected from the loads and stresses at the critical locations can be used to transfer fatigue tests. If there are any changes in a critical location, a new transfer function will be required to accommodate the changes. The advantages of the IAT program are as follows:

- It gives insight into actual usage compared to the design envelope of each aircraft.
- With the load monitoring of the primary structure and the fatigue test result, fatigue life approximation of the critical components of each aircraft can be made or their damage status determined.
- Fatigue life approximation translates into a scheduled maintenance plan.
- The operation can be altered to control the rate of fatigue life usage.
- A database on operational load and flight tests can be compiled to implement fatigue tests and comparison with initial fatigue test data.
- The discrepancy of the fleet-wide operational condition of aircraft can be determined by considering mission intensity, storage condition, and point-in-the-sky effects.
- Better insight can be gained into the loading environment through flight test data.

### 1.8. Structure Life Extension 

There may come a point when one must consider a life improvement program to either overcome deficiencies that arise during planned service or extend operation beyond the original life goal. The first step is to perform a new damage tolerance analysis that considers prior incidents of fatigue or corrosion, the actual repair and maintenance history, and the desire for future usages, such as projected load histories. If the structure were initially damaged, the new study would confirm whether sufficient protection was provided to fracture-critical areas and whether existing maintenance plans are adequate. If the structure was not designed to damage the tolerant principle, this analysis is essential to identify potential areas of future vulnerability [15]. The new analysis should employ the latest NDT method, reflect the most recent structural condition assessment, account for potential modifications or repairs, and consider project usage. 

The Finnish Air Force (FAF) did an excellent job of extending the ultimate wing usage life of the Hawk Mk 51 [16], ensuring that the wing’s main structure is still under the safe-life condition. The result is used for engineering judgment to extend the wing service life of a particular aircraft fleet possibly. The study was initiated when the FAF discovered that the actual aircraft mass was 4700 kg, more than the design weight of 4400 kg. In addition, cracks were found in the wing’s main structure during the fatigue test. 

A full-scale fatigue test (FSFT) enables more accurate structural damage detection and increases prediction service life accuracy. Based on the findings from the fatigue test, a more realistic and accurate service life is predicted. The execution is compliant with MIL-STD-1629A and MIL-STD-1530C. However, the FSFT is not compulsory due to its high cost and complex system; a finite element analysis (FEA) is a more reasonable alternative. 

The Polish Air Force (PAF) implemented a similar approach, which introduced the structural integrity program called the SEWST, developed by Airbus Military in cooperation with the Air Force Institute of Technology (AFIT) for the PZL-130 Orlik TC-II military trainer aircraft. The aircraft was developed in the 1980s and was put into service as a TC-I version in 1994. Due to constant improvement, a TC-II version was designed mainly with modernized wings (a larger wing area and wingspan), an overhauled and strengthened fuselage with larger empennage, and modernized avionics. Since the new version of the aircraft is to be operated for a prolonged time (expected service life of 12,000 flight hours), a modern structural integrity program was developed based on the Aircraft Structural Integrity Program (ASIP), the NDT, and MIL-STD-1530C.

The ASIP approach in the SLEP has been used and adopted by most air forces. For example, the USAF has extended the life of KC-135 Stratotanker up to an incredible 80 years or more from its original date of induction into service [17]. The same goes for Canada’s Sea King helicopters (1963 to 1969). These helicopters have accumulated up to 10,000 to 12,000 flying hours. Unfortunately, they have many repair patches on structural members. During the planned upgrade, some of these structural members are envisaged to be replaced [18], and the Australian fighter aircraft F-111s, which were first introduced in the United States in 1976, will probably get phased out. However, the RAAF is acquiring these aircraft, and after upgrading, they plan to use them until 2022 [19], all thanks to the NDT structural assessment.

In another development, the given whole life of 1500/15 years of Indian Air Force (IAF) fighter aircraft was extended to 2500 h/25 years through its life extension program with the help of various R&D studies; the public sector; and academic, certification, and inspection agencies under the project code-named Project LIFEX using latest NDT techniques [20]. 

As a result, it was estimated that out of a population of 6000 old fighter aircraft worldwide, nearly 3000 would be used with an extended life span.

### 1.9. Maintenance Task Card for Structural Assessment

RMAF aircraft maintenance processes are regulated by the Directorate General of Technical Airworthiness (DGTA) stipulated in the Technical Airworthiness Management Manual (TAMM) regulation 3.5.15. The Aircraft Structural Integrity Management approach is used for the development of the maintenance task card. Several elements must be established to form the Aircraft Structural Integrity (ASI) management system. There are two main principles. First is the inspection manual that encompasses the NDT as its fundamental element. The second component is the prediction analysis, which is engineering analysis through FEA simulation with supplementary data from structure health monitoring (SHM) and the flight data recorder (FDR) for the loading profile. 

In this case, the change involved in the Instruction of Continuing Airworthiness is developing the new maintenance task card. This involves changes to the pre-existing maintenance and repair standard, while maintaining the aircraft design specification. This requires technical approval from the DGTA as the regulatory body. The document produced under this program is 10-year servicing of the Su-30MKM task card and 10-year servicing of Su-30MKM technical tasks. 

### 1.10. Non-Destructive Testing Method for Structure Life Extension

Environment degradation is one of the main factors that significantly compromise the mechanical properties of the aircraft structure and components. For example, environmental conditions, such as high temperature and humidity, can cause severe corrosion, compromising the integrity of the metallic structure. UV radiation and pollutants in the atmosphere can also degrade the structure of non-metallic components, such as composites and polymers of the aircraft components. Therefore, the emerging non-destructive testing (NDT) method is widely adopted for in-depth inspection and monitoring of the aircraft structure. NDT also involves the categorization of defects. Early detection is crucial for preventing catastrophic failure. NDT has become relevant in the detection of in-service defects as well as assessing the integrity of the structure with various techniques, such as ultrasonic testing, liquid penetrant testing, eddy current testing, radiography, and the magnetic particle method, depending on the specimen type. 

The Sukhoi aircraft based on the safe-life fatigue design philosophy has an optimum loading factor. Besides that, the aircraft’s service life is also based on its calendar due date and landing cycle. In the case of RMAF Su-30MKM, the calendar due date has arrived and structural assessment for life extension is required. According to Srinivasan and Vijayaraghavan [21], the fatigue index (FI) of each aircraft is calculated by taking readings from a fatigue meter. Apart from the fatigue life, NDT is also used to check the structural condition of various components. Consequent to the NDT inspection and the results obtained, designers from the OEM have agreed to increase the existing structural life of the aircraft in terms of its FI and calendar life.

## 2. Methodology

The main methodology of this structure life extension program is the use of non-destructive inspection (NDI), which takes advantage of NDT-certified Level III experts in the RMAF with the certification standard of EN4179/NAS410. In addition, finite element analysis is performed to determine the fatigue life of the critical components of the aircraft. 

### 2.1. Fighter Aircraft Fatigue-Critical Location 

Currently, the RMAF Sukhoi is not fitted with any fatigue-measuring sensors. The Sukhoi is designed using the safe-life fatigue design spectrum, with its ultimate life based on either the loading factor or its flying hours. The critical locations on the aircraft structure are identified based on its load-bearing members. A critical location is defined as the part of an aircraft that is prohibited from having any defect. It is highly possible that any defect in that part will lead to catastrophic failure and be life threatening. The critical locations are as follows:

- Wing root
- Vertical stabilizer attachment
- Horizontal stabilizer attachment
- Engine mounting
- Canard attachment
- Fuselage attachment

### 2.2. Non-Destructive Evaluation

The discontinuities on a structure can be on its surface or sub-surface, the defects spanning welded joints, pores, micro-cracks, inclusion, residual stress site, and microstructural degradation. Action must be taken on the detected discontinuities, and the structure must be either replaced or repaired; neglecting this will ultimately cause severe structural damage and failure. While the defect should be addressed and repaired immediately, the crack rate can be monitored with a sound understanding of the characteristics of the crack (its size and location) and the local properties of the material. 

**Figure 3:**
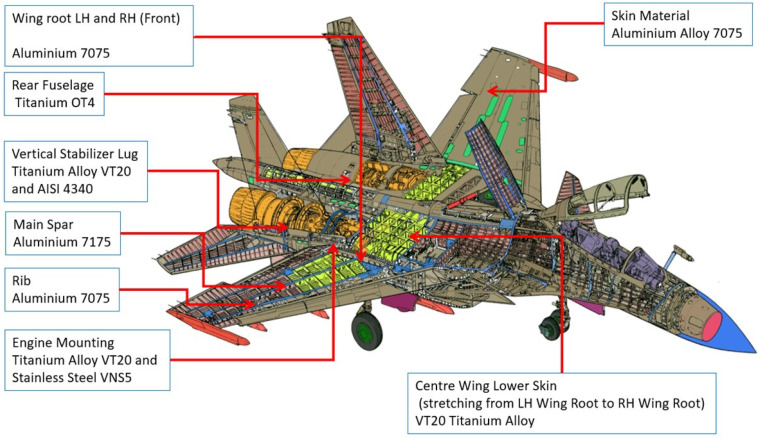
The material composition of the Su-30MKM structure.

Before performing NDT, the material composition of each critical structure is identified. This will aid in the determination of a suitable method that will be used to inspect that particular area. The material composition of the structure is depicted in Figure 3. There are no control or regulatory samples used in this analysis other than the calibration samples for each method provided with the test equipment.

Fatigue failure is a gradual form of local damage determined by the magnitude and frequency of the loads introduced on the element. Aircraft structures or components are inevitably subjected to fluctuating stress and, hence, prone to defect or crack initiation, leading to failure by fatigue fracture. The NDI for Su-30MKM PRW is divided into 3 main sections. The NDT inspection instruction was developed by the RMAF Central Aerospace and Engineering Services Establishment engineers who are Level III qualified with EN4179/NAS410 standards. 

#### 2.2.1. Liquid Penetrant Inspection

This method is suitable for hard-to-reach surface area and crevices. Liquid penetrant inspection uses the advantage of a liquid’s properties, which can coat the aircraft pins, attachment, brackets, fuselage ailerons, wings, and engine mounting. This method can be used on all ferrous and non-ferrous components. The principle of LPI is that LPI generally uses a fluorescent penetrant, increasing the contrast of the discontinuity to be detected via visual inspection. Therefore, this method can detect discontinuities from corrosion. The most common liquid penetrant is Magnaflux ZL-27A (the developer is SKD-S2, and the remover is SKC-S), with a standard dwell time of 15 min. In this inspection, the method used was the solvent remover method with a sensitivity level of 4. In addition, the penetrant (Magnaflux ZL-27A), developer (SKD-S2), and remover (SKC-S) were used. The inspection was carried out based on the instructions provided.

#### 2.2.2. Magnetic Particle Inspection

The magnetic particle inspection (MPI) method, suitable for steel and ferromagnetic materials, can detect surface and close-to-surface discontinuities. These leakage fields are monitored using a prepared bath spray of fine particles in the fluorescent penetrant sprayed on the magnetized component using an AC Yoke. The components inspected using this method were the engine-mounting trunnion, lock bolts, the horizontal stabilizer pivot, and struts. These components were inspected using the MPI method since they were solid structures and their load paths or failure points would be on their surface. The inspection was performed using the Magkraft AC Yoke (MK-30-120-B) and the Magnaflux 14AM aerosol.

#### 2.2.3. Eddy Current Inspection (ET)

Generally, an eddy current inspection, as the name suggests, takes advantage of the magnetic field generated by an eddy current. The ET method works with all ferrous materials. These include the main fuselage bulkheads, engine mounting, wing attachments, and joints. A trained inspector can detect both surface and sub-surface discontinuities. The most common on-market ET equipment is the Olympus Nortec 600D (S/No: 60012173886) with an angle probe with a frequency of 300 KHz. This equipment was used in this inspection with an angle probe with an appropriate frequency. A high-frequency inspection was performed to determine any sub-surface discontinuity. The calibration block used was of aluminum alloy with a conductivity of 31.49%IACS.

#### 2.2.4. Radiography Inspection

Radiography inspection (RT) is one of the effective methods of NDT for aircraft components and structures. The principle of operation is relatively straightforward. Similar to the X-rays used in the medical field, this method uses gamma rays to penetrate the specimen. Discontinuities will be generated in the images as the gamma rays penetrate the specimen. This inspection was carried out using the Dandong Aolong, Model XXG-2505 (S/No:80457) radiography set. The inspected components were engine inner and outer trunnion mounting; canard shaft endpins; outer wing front, center, rear spar; and horizontal stabilizer front and rear spar.

## 3. Results and Discussion

### 3.1. Non-Destructive Testing Results

**Figure 4:**
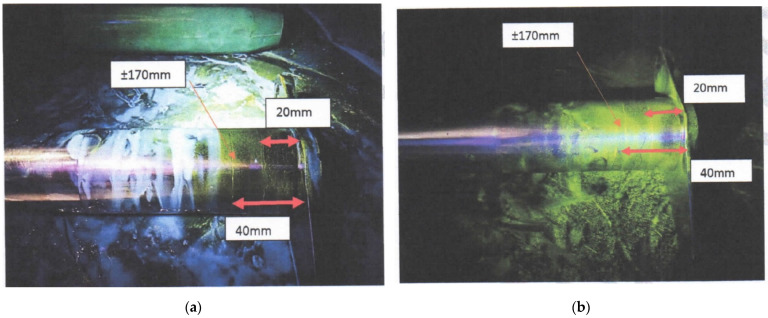
Discontinuity detection by the MPI method. (**a**) LH engine mounting (MPI). (**b**) RH engine mounting (MPI).

Table 3 depicts the results of the NDT inspection that was carried out on the FCL of the aircraft. For simplicity, Figure 4 shows only those instances where cracks were found.

### 3.2. Fatigue-Critical Location Assessment

**Figure 5:**
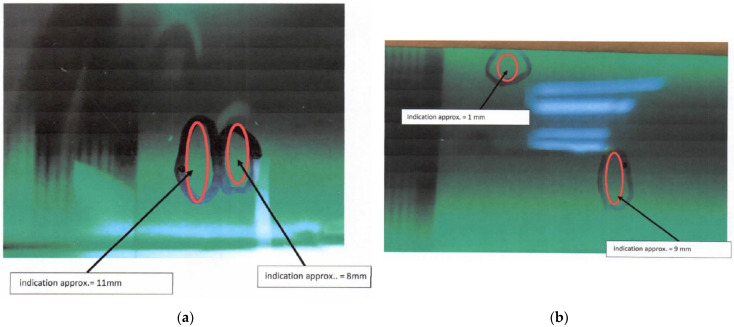
Discontinuity detection using the RT method. (**a**) RH engine mounting (RT). (**b**) LH engine mounting (RT).

Comprehensive inspection of all FCLs showed discontinuities on assembly parts on the LH and RH engine mounting, exposed by both MPI and RT methods. Detailed images of the defect are depicted in Figure 4 and Figure 5. The discontinuity detected on the engine mounting was likely due to the vibration caused by the engine during operation. This continuous loading caused a crack initiation, which further propagated to the surface as a defect. However, it did not propagate further internally and cause catastrophic damage. 

The aircraft has been in service for more than 10 years; the justification for extending the aircraft’s service life is based on the assessment and inspection conducted via NDI. Based on the inspection, the other FCL indicates no damage inflicted by fatigue stress or operation beyond the limitation of the design envelop. There is no defect in the critical structure of the aircraft that could jeopardize the airworthiness of the aircraft. 

### 3.3. Critical Component Computer-Aided Design (CAD) Model

**Figure 6:**
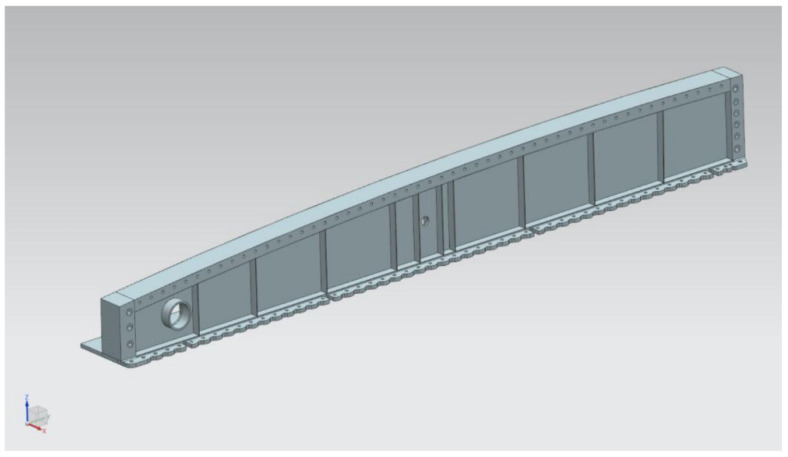
Wing root CAD model.

The wing root, one of the critical components, was chosen to be analyzed using FEA for fatigue calculation. This component was chosen since it is the location that is the highest load-bearing member. The wing root is attached directly to the airframe of the center wing. The lower part of the wing root was chosen to be the crack initiation location. The wing root structure was chosen among the critical/primary locations because it functions as an attachment between the outer and center wings. This location is the concentration point for wing root bending moment. As shown in Figure 6, the original 3D CAD model was produced from a 3D scanning process. CAD development started with working on the wing root CAD model. The dimension and design of the model were obtained using the 3D scanning method. The scanned 3D CAD model went through a cleaning process and surfaces representing spars, longerons, frames, and bulkheads before it could be suitably used for CAD analysis.

#### 3.3.1. Material Properties and Boundary Conditions for FEA

**Figure 7:**
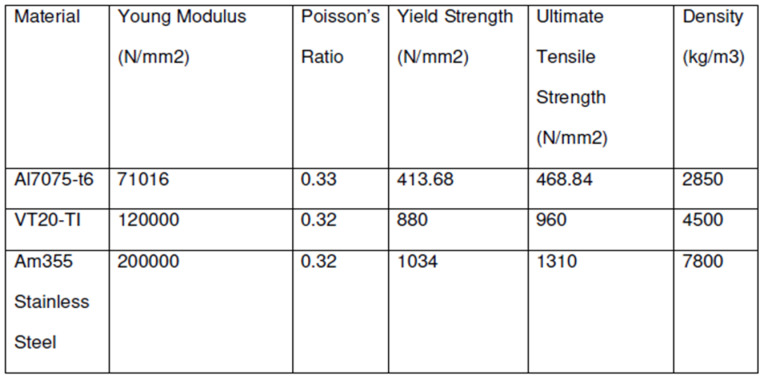
Material properties for the wing root joint.

**Figure 8:**
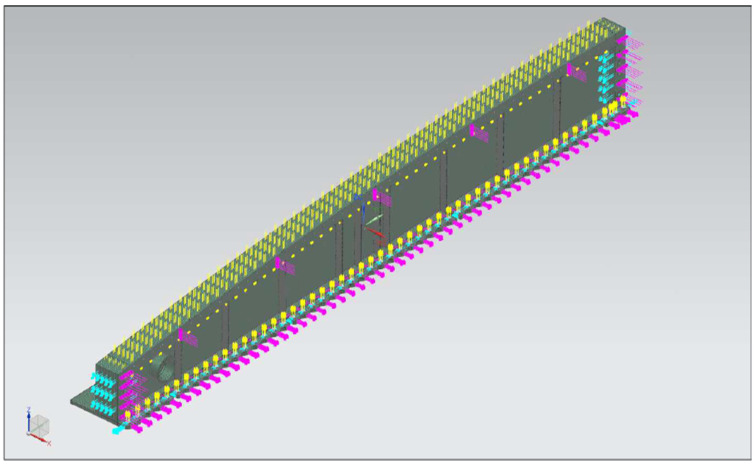
Boundary conditions of wing root joint FEA.

The RMAF made initial material identification in collaboration with the Science and Technology Research Institute for Defence (STRIDE) material-scanning capability, and additional material properties were obtained from the Metallic Materials Properties Development and Standardization (MMPDS) handbook and the Titanium Alloy Russian Aircraft and Aerospace Application [22]. The material identified by the RMAF and STRIDE is the VT20 titanium alloy, and the properties were referred to from [23]. The primary material properties used are depicted in Figure 7, and the boundary conditions are depicted in Figure 8. 

Boundary conditions for the FEA global model are a tricky modeling situation. The aircraft is flying at a steady level flight, all its control surfaces at their default positions. This entails that none of the aircraft bodies be bound to the environment in any FEA ways; therefore, the inertial relief function was used. Inertial relief automatically applies inertia forces distributed to all nodes of the model to form an equivalent state of static equilibrium. Inertial relief is useful if the body being analyzed cannot be constrained in any way. Without inertial relief, the body analyzed statically cannot be solved as it has rigid body motion. Loading profiles consist of pressure profiles of aircraft due to cruising speed and altitude, imported into the FEA global model and pressure profiles on the aircraft due to fuel weight.

**Figure 9:**
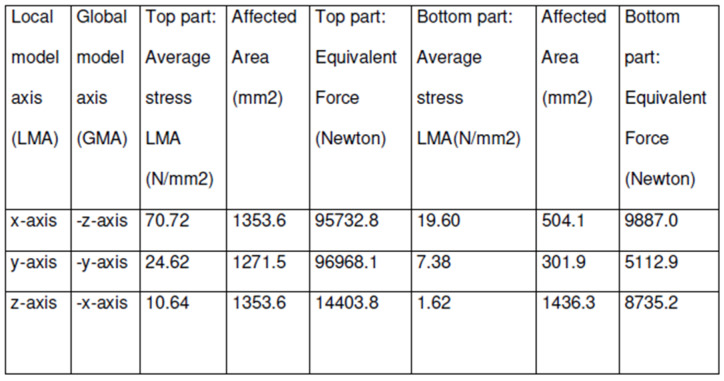
FEA loading table.

The loading and the forces applied to the wing lug were extracted from the FEA global model. The extracted stresses, forces, and their corresponding axes are displayed in Figure 9.

#### 3.3.2. FEA on the Local Model 

**Figure 10:**
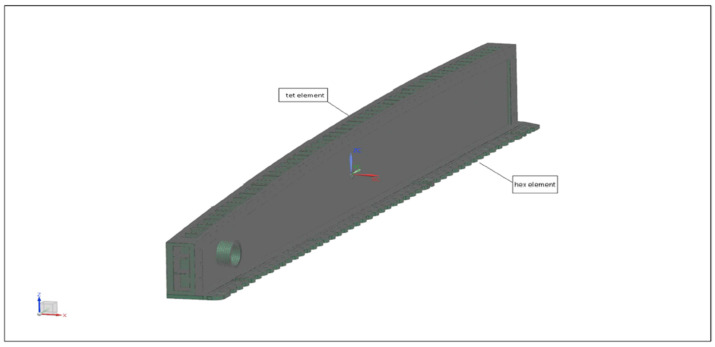
Meshing of the FEA model.

Static analysis was performed on a wing root model using N.X Nastran software. The wing root geometry was divided into two smaller parts. This was done due to these parts having different material properties. Figure 10 shows the FEA local model. The parts and their elements are connected using the surface-to-surface glue method. 

#### 3.3.3. Fatigue Analysis Results 

The fatigue life calculation of Sukhoi Su-30MKM was performed on the wing–fuselage lug joint structure. The wing lug comprises two blocks, which are made using Al7075-T6 (aluminum alloy) and VT20-TI (titanium alloy). 

**Figure 11:**
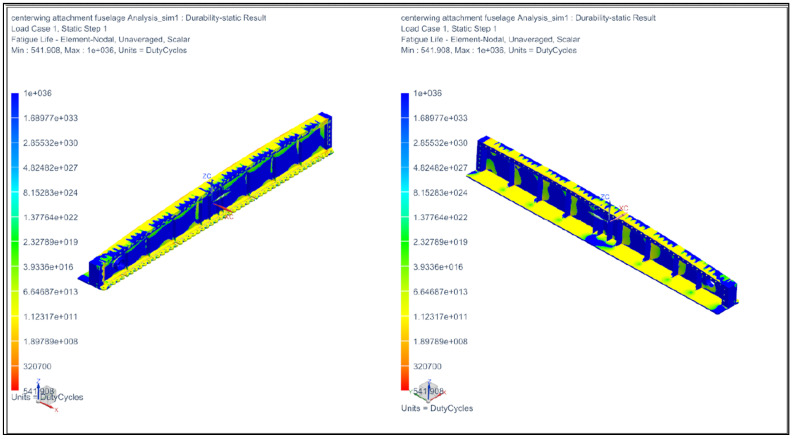
Finite Element Analysis of the wing root joint.

As shown in Figure 11, the damage value of 0.0018 shows that only 0.18% percentage of the damage occurred on the wing lug. Note that the value of 1 means that the damage was 100%. In addition, from the result, a cycle to failure of 541 cycles means it is safe in terms of fatigue since one cycle is about 10 years’ worth of flying. Therefore, it will take 541 × 10 years per cycle for the wing root to fail. The “local model stress result” was expected from the static analysis that fatigue failure would not happen, and the fatigue result confirmed this observation. FEA regional analysis shows that the stress level at the wing root of 193.36 MPa is acceptable and the wing root can be deemed safe and adequately designed for mechanical static scenarios. A quick analysis shows that this value is 20.1% of the ultimate tensile strength of VT-20-TI, and since the value is below 40% of the UTS, it is expected that fatigue failure is unlikely for the wing root. The explanation is tabulated in Table 4 below by years of Su-30MKM service. 

### 3.4. Model Validation with Experimental Data

The crack growth model had to be validated. The prediction was compared with given data in [23,24,25]. The crack growth data were tested under variable loading blocks using 7075-T6 aluminum alloy in this study. 

#### 3.4.1. Crack Growth Rate Constants of Specified Materials

The specified material crack growth constant should be determined before any calculation is conducted. Since data were lacking, data from [24,25] were used to determine the crack growth constant. The fatigue growth data from other papers were used for AL 7075-T6. Initially, R = −0.8 was used for the loading spectrum. For this validation, R = −1, 0, and 0.5 were used for the crack growth data. The necessary crack growth data were rescaled using equations with the given values of β = 0.7 and $\beta_{1}$ = 0.84. Taking into account the geometrical parameters of the specimens, the parameters are listed in Table 5. The experiment should determine the shaping parameter *n*. However, the value of *n* would be different from the predicted result and could cause misunderstanding when the data are used to predict the fatigue life subjected to variable loading. For simplicity, the shaping exponent *n* was set to the same value with the same material.

#### 3.4.2. Predicted Result Compared with Experiment Data

**Figure 12:**
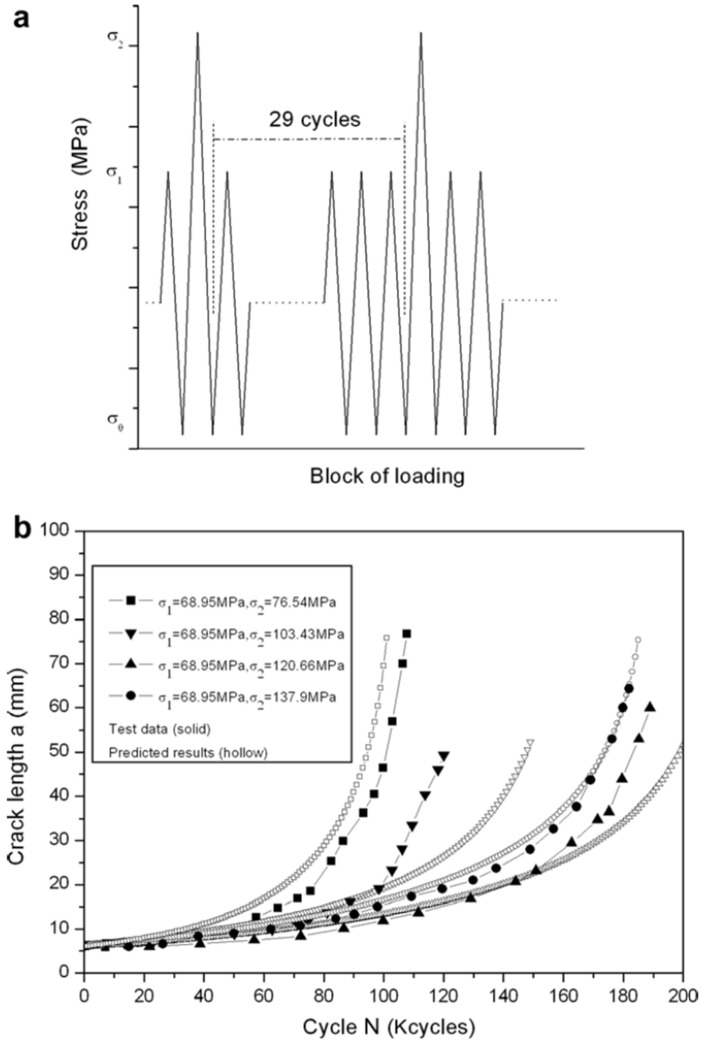
Comparison of AL7075-T6 test data [26]. (**a**) Loading blocks and (**b**) comparison of the predicted model with experimental data.

The crack growth data of the material were subjected to variable loading by Porter [26]. This study was used to validate the current crack model. Figure 12a shows the loading block. Figure 12b compares the AL7075-T6 crack growth model with different *p*, *q* values. The prediction model was compared with test data shown in Table 4. The curve could be seen as abnormal. The current model was used for the prediction of crack growth under variable cyclic loading, as mentioned. Crack models such as NASTRAN and AFGROW were used. It was concluded that different loading blocks yield different results, while the crack growth model still provided valid results, as depicted in Figure 12b [27]. 

## 4. Conclusions

The ASI activity data collection for Sukhoi Su-30MKM can shed more light on the current fatigue condition of the aircraft’s critical locations. Based upon loading factor data, the overall loading factor is 2,065,386 out of 21,000,000. This ratio of 0.098 (9.8%) means the damage on the whole aircraft is less than 10% in terms of the loading factor. The methods for calculating the loading factor and fatigue analysis are not similar. Even though ASI data and the fatigue result are not comparable, it could shed light on whether the damage due to fatigue is distributed across the aircraft. Therefore, fatigue analysis is recommended for other locations that are deemed critical. For fatigue analysis, the loading profile for each year was set up according to year. Therefore, the damage contribution for each year can be extracted.

The results also showed that with stress loading amounting to almost 9G, the maximum force acting on the structure was lower than the maximum yield strength. This can provide a solid basis for the extension of the wing root for the aircraft. In addition, the Aircraft Structural Integrity Program initiated for the fleet will further enhance the structure assessment through condition and usage monitoring.

The aircraft aging process through wear and tear is inevitable; however, a comprehensive engineering approach can be implemented to manage and minimize the effect of the aging process. An in-depth understanding is crucial to mitigate the deterioration of the structural integrity of the aircraft. The justification and judgment for service life extension of said aircraft must be backed by sound engineering analysis and procedures. Preventive maintenances procedures should be streamlined to the need of the aging aircraft. Procedures such as the NDI method are recommended to be performed periodically and adequately. 

Su-30MKM aircraft operate in tropical environments with high temperatures and humidity, considered adverse conditions for the aircraft structure. However, the manufacturing process is proven to be excellent; in the study, only a specific location of the surface was found to have become defective. The defects found in the various location are within the safety design limit. The engine mounting is suggested to be replaced during each overhaul, depending on the NDT assessment of the component. In addition, all of the FCLs are suggested to be further analyzed using crack growth prediction to further detail the structure usage limitation in the case of structural damage. The service life extension is justified based on the NDI method conducted and validated through the fatigue simulation analysis of the Su-30MKM critical components. In addition, this study is not based on durability and damage tolerance assessment but on the safe-life concept, which is the fatigue design philosophy of this aircraft. 

## Figures and Tables

**Table 2:** Summary of Structure Life Extension Research.

No	Author	Topic	Concept	Total Number of Years Extended	Analysis Used
1	Kurnyta et al.	Ageing Fighter—Bomber Aircraft Durability Tests and Operational Load Monitoring to Support Life Extension Program	1. Ground and flight tests for a separate aircraft. 2. FDR archive analysis for 5 years of operational use of the whole fleet. 3. Full-scale durability test for the retired aircraft. 4. Operational loads monitoring (OLM) system implementation for all Su-22UM3K.		Full-scale fatigue test
2	Ilic et al. (2011)	Important Aspects for Extension of Fighter Plane Service Life by Performing Overhaul Based on the Actual Condition	1. Analysis of the plane documentation for information such as flight hours, working conditions, frequency of conducting established preventive maintenance, flight profiles, typical failures, and their consequences for the plane. 2. Overhaul based on its actual condition, which means that the aircraft components will be replaced as required. This means that the majority of the components that have failures will be replaced, which can extend the life of the aircraft.		Document history and modification
3	Clark	Aircraft Fatigue Life Extension: Development of a Mid-life Rework Method Based on Peening	1. Rework method in which a surface layer that has been damaged by cracking, or by other methods, is removed in a controlled manner, a crucial factor when reworking critical parts where a small error can lead to extremely expensive repairs. The clean surface can then be peened using a controlled technique that produces minimal damage on the surface and maximizes the life improvement factor (LIF) for the process.		Modification to critical components
4	Pentz (2000)	A Service Life Extension (SLEP) Approach to Operating Aging Aircraft Beyond Their Original Design Lives	1. Aircraft mission data collection and simulation model. 2. Accuracy of the simulation model. 3. Testing the validity of the simulation model. 4. Planning simulations to be performed. 5. Conducting the simulation and data analysis.	600 to 2000 h	Engineering analysis
5	Molent and Aktepe (2000)	Review of Fatigue Monitoring of Agile Military Aircraft	1. Fitment of airborne fatigue monitoring system. 2. Individual aircraft fatigue monitoring system. 3. Operational load monitoring to determine the loads at the critical locations compared to g-meters. 4. Usage monitoring data based on flight hours, mission type, duration, and aircraft weight data.	2000 h	On-board sensors
6	Maksimović et al. (2015)	Service Life Extension Program for Aircraft Structures	1. Non-destructive inspection methods. 2. Numerical structure analysis (stress analysis from the aspect of fatigue and fracture mechanics). 3. Simulation analysis (flight loads and load cycle analysis). 4. Experimental testing of aging structure specimens. 5. Software development for the prediction of remaining service life beyond the designed service life. 6. The methods are used for the prediction of the remaining service life of the aircraft beyond its designed service life.	1000 h	Computation and experiment
7	Rui et al. (2018)	Individual Aircraft Life Monitoring: An Engineering Approach for Fatigue Damage Evaluation	1. Mechanical properties of related materials are obtained, including the elasticity modulus, the yield strength, the tensile strength, and the *S–N* curve. 2. The load spectra for full-scale fatigue tests are definitely determined. During the aircraft structural development stage, the load spectra for full-scale fatigue tests are compiled based on relevant theories and experience, and the load spectrum for each critical structure can therefore be obtained. 3. Regarding the fatigue life of aircraft structures, full-scale fatigue test data are complete and comprehensive. Full-scale fatigue tests are conducted under the predetermined load spectrum to identify critical locations and obtain pertinent crack growth information during the final stage of aircraft structural design. 4. Realistic load spectra for individual aircraft can be developed based on the operational data.		Traditional nominal stress method
8	Desai (2001)	Life Extension of Aircraft Components—An IAF Perspective	1. Assessment of structural condition and timely detection of structural problems. 2. Fatigue testing and teardown inspection. 3. Improved NDE technology.	2500 h/25 years	Non-destructive testing
9	Molent et al. (2012)	Verification of an Individual Aircraft Fatigue Monitoring System	1. Using an enhanced teardown procedure, in-service fatigue crack growth was identified at a significant number of locations. All the in-service cracking corresponded to the same locations found cracked in the fatigue certification full-scale test article that was used to calibrate the usage monitoring system. By comparing the measured in-service growth with the test-demonstrated growth, the functionality of the monitoring system could be assessed. This assessment should reveal the system’s effectiveness in providing robust fatigue-life-expended indices to help ensure that structural integrity boundaries are not exceeded. For this comparison, the crack growth was measured using quantitative fractography.		Previous condition compared with a simulated test

**Table 3:** NDT inspection result.

Wing Root LH and RH	
**Method**	**Result**
Eddy current inspection	High-frequency eddy current inspection was carried out, and NIL crack indication was found.
Liquid penetrant inspection	Fluorescent penetrant inspection was carried out, and NIL crack indication was found.
**Vertical Stabilizer LH and RH Attachment**	
Eddy current inspection	Eddy current inspection was carried out, and NIL crack indication was found.
**Horizontal Stabilizer LH and RH Attachment**	
Magnetic particle inspection	Magnetic particle inspection was carried out, and NIL crack indication was found.
**Engine Mounting LH and RH**	
Magnetic particle inspection	Magnetic particle inspection was carried out and discontinuities were found at the external and internal surface of the mounting, as depicted in Figure 4.
Radiography inspection	X-ray inspection was carried out on the engine mounting and discontinuity was found, as depicted in Figure 5.
**Canard LH and RH Attachment**	
Eddy current inspection	Eddy current inspection was carried out, and NIL crack indication was found.
**Upper Longeron at Frame No. 18**	
Eddy current inspection	Eddy current inspection was carried out, and NIL crack indication was found.
**Center-rear attachment at Frame No. 34**	
Eddy current inspection	Eddy current inspection was carried out, and NIL crack indication was found.

**Table 4:** FEA loading table by years.

Year	Damage
2008	3.32 × 10^−5^
2009	0.00021
2010	1.99 × 10^−5^
2011	0.00029
2012	0.00011
2013	0.00040
2015	0.00069
2017	8.86 × 10^–5^
TOTAL	0.0018

**Table 5:** Geometry and material variation in specimens.

Specimen Material	*σ_y_* (MPa)	*C*	*m*	*n*	*t* (mm)	*w* (mm)
7075-T6 (aluminum alloy)	520	6.85 × 10^−8^	3.21	0.3	4.1	305
2024-T3 aluminum alloy	315	3.0 × 10^−8^	3.1/3.2	0.32	4.1	229
350WT steel	350	1.5 × 10^−8^	2.8	0.5	5	100

## Data Availability

The data presented in this study are available on request from the corresponding author.

## References

[1] Reddy S.K., Laboratories I.R.S.N. (2017). Fatigue Failure of Aircraft Component.

[2] Wilson E.S. Development RAAF Aircraft Structure Integrity Management. Proceedings of the 18th Symposium of the International Comittee ion Aeronautical Fatigue: Estimation, Enhancement and Control Aircraft Fatigue Performance.

[3] Christopher L. (2020). Effect of Cold Work Expansion on the Fatigue Life of Pre-Cracked Al 2024-T3 (2020). Ph.D. Dissertations and Master’s Theses. https://commons.erau.edu/edt/547.

[4] Negaard G.R. (1980). The History of the Aircraft Sructural Integrity Program.

[5] Venugopal A., Mohammad R., Koslan M.F.S., Sayd Bakar S.R., Ali A. (2021). The Effect of Tropical Environment on Fatigue Failure in Royal Malaysian Airforce (RMAF) Aircraft Structure and Operational Readiness. Materials.

[6] Byron R.A. (1981). Fatigue Aircraft.

[7] Heida J.H., National Aerospace Laboratory (1998). Airframe Inspection Reliability Using Field Inspection Data.

[8] Liao M., Renaud G. (2010). Fatigue Analysis for CF-18 Component: Wing Fold Shear-Tie Lug.

[9] Kejuruteraan B., RMAF (2007). Progressive Maintenance. Technical Equipment Maitenance Plan Su-30MKM.

[10] USAF (2004). MIL-STD-1530C Aircraft Structure Integrity Program (ASIP).

[11] Kyu P.J., Han L.D., Jae L.C., Cheul C.B., Tae C.K. (2001). Finite Element Method Analysis and Life Estimation of Aircraft Structure Fatigue/Fracture Critical Location.

[12] Jonge J.B.D. The monitoring of fatigue loads. Proceedings of the 7th Congress of the International Council of Aeronautical Sciences.

[13] Crocker D., Oore M. Individual aircraft tracking system for the Canadian P-3C (CP-140/A) aircraft structural integrity program. Proceedings of the 18th Symposium of the International Committee on Aeronautical Fatigue: Estimation, Enhancement and Control of Aircraft Fatigue Performance.

[14] Molent L. (1997). A Review of a Strain and Flight Parameter Data Based Aircraft Fatigue Usage Monitoring System. USAF ASIP Conference.

[15] Grandt A.F. (2004). Fundamentals of Structural Integrity: Damage Tolerence Design and Nondesreuctive Evaluation.

[16] Patria J.T. Fatigue Life Evaluation of Critical Locations in Aircraft Structures Using Virtual Fatigue Test. Proceedings of the International Congress of The Aeronautical Sciences.

[17] USAF (2013). USAF Complete Evaluation of KC-135 Block 45 Upgrade.

[18] Brewster M. (2015). Sea King Helicopter to Remain in Skies Past Retirement. CBC News Nova Scotia.

[19] Goon C.K., Defence D.O. (2005). Air Power Australia.

[20] Desai P.K., Raganath S.T.V.R. (2001). Life Extension of Aircraft Component-An IAF Perspective. Recent Trends in Structural Integrity Assessment.

[21] Srinivasan M., Vijayaraghavan P. (2015). NDE for Prevention of Failures and Life Extension of Structures and Components of Aircrafts.

[22] Moiseyev V.N. (2006). Titanium Alloys Russian Aircraft and Aerospace Applications.

[23] Yarema S.Y., Grechko V.V., Ostash O.P. (1978). Cyclic Crack Resistance to VT20 Titanium Alloy Sheets and Its Anisotropy. Fiz. Mekhanika Mater..

[24] Taheri F., Trask D., Pegg N. (2003). Experimental and analytical investigation of fatigue characteristics of 350WT steel under constant and variable amplitude loading. Mar. Struct..

[25] Ray A., Patankar R. (2001). Fatigue crack growth under variable-amplitude loading: Part II-Code development and model validation. Appl. Math. Model..

[26] Porter T.R. (1972). Method of analysis and prediction for variable amplitude fatigue crack growth. Eng. Fract. Mech..

[27] Venugopal A., Mohammad R., Koslan M.F.S., Shafie A., Ali A.B., Eugene O. (2021). Crack Growth Prediction on Critical Component for Structure Life Extension of Royal Malaysian Air Force (RMAF) Sukhoi Su-30MKM. Metals.

